# Knockdown resistance of *Anopheles sinensis* in Henan province, China

**DOI:** 10.1186/s12936-015-0662-y

**Published:** 2015-03-31

**Authors:** Hong-wei Zhang, Ying Liu, Tao Hu, Rui-min Zhou, Jian-she Chen, Dan Qian, Cheng-yun Yang, Yu-ling Zhao, Su-hua Li, Jing Cui, Zhong-quan Wang, Zhanchun Feng, Bian-li Xu

**Affiliations:** Department of Parasite Disease Control and Prevention, Henan Center for Disease Control and Prevention, Zhengzhou, 450016 P. R. China; School of Medicine and Health Management, Tongji Medical College, Huazhong University of Science & Technology, Wuhan, Hubei 430030 P. R. China; Department of Parasitology, Medical College, Zhengzhou University, Zhengzhou, 450052 P. R. China

**Keywords:** *Anopheles sinensis*, Knockdown resistance, *kdr* mutation, Henan province

## Abstract

**Background:**

Vivax malaria was historically epidemic in Henan Province of China and *Anopheles sinensis* was the main vectors and poor farming communities bare the greatest burden of disease. Knockdown resistance in *An. sinensis* is one of the mechanisms of resistance against pyrethroids. In the present study, the frequency of mutations from *An. sinensis* was examined in Henan province, China.

**Methods:**

Anopheles was collected from Kaifeng, Tongbai, Tanghe, Pingqiao, Shihe, and Yongcheng counties of Henan province in 2013. Molecular identification of Anopheles species was conducted by polymerase chain reaction (PCR) amplifying the internal transcribed spacer 2 (ITS2). Part of the IIS6 domain of the para-type sodium channel protein gene was polymerase chain reaction-amplified and directly sequenced. Frequency and geographic difference of *kdr* gene mutant types were analysed.

**Results:**

208 Anopheles were received molecular identification, of which 169 (81.25%) were *An. sinensis*, 25 (12.02%) were *Anopheles yatsushiroensis*, and 12 (5.77%) were *Anopheles lesteri*. A 325 bp fragment of the para-type sodium channel gene including position 1014 was successfully sequenced from 139 Anopheles, of which 125 (89.93%) were *An. sinensis*, 12 (8.63%) were *An. yatsushiroensis*, 2 (1.44%) were *An. lesteri*. The molecular analyses revealed that mutations existed at codon 1014 in *An. sinensis* but not in *An. yatsushiroensis* and *An. lesteri*. Frequency of *kdr* mutation was 73.60% (92/125) from population of *An. sinensis* in Henan province, of which L1014F (TTT + TTC) allele frequencies accounted for 46.40% (58/125), and was higher than that of L1014C(TGT) which accounted for 27.20% (34/125) ( *χ2* = 55.423, *P <* 0.001). The frequency of *kdr* mutation in Kaifeng county was 100% (49/49), and was higher than that of 37.93% (11/29) in Tongbai, 54.55% (6/11) in Pingqiao, 50.00% (3/3) in Shihe, and 62.50% (10/16) in Yongcheng county, respectively (*χ2* = 39.538, *P <* 0.001; *χ2* = 24.298, *P <* 0.001; *χ2* = 25.913, *P <* 0.001; *χ2* = 20.244, *P <* 0.001). While 92.86% (13/14) frequency of *kdr* mutation was found in Tanghe county, which was higher than that in Tongbai county (*χ2* = 11.550, *P* = 0.0018).

**Conclusions:**

A high frequency of *kdr* gene mutations from population of *An. sinensis* in Henan province was found.

## Background

*Anopheles sinensis* is the main vivax malaria vectors and poor farming communities bear the greatest burden of disease in China and other Southeast Asian countries [1-4]. Indoor residual spraying and long-lasting insecticidal nets are recommend by the World Health Organization (WHO) as effective vector control measures to prevent malaria transmission [5,6]. Nowadays, pyrethroids are emerging as the predominant insecticides for vector control because of their low toxicity to humans, high efficacy against mosquito vectors and short residual action. In the past decade, pyrethroids have become the preferred choice among the currently WHO approved compounds. 414 tonnes of pyrethroids were used annually for global vector control during the period 2000–2009 in the world. 68% (282/414 tonnes) of pyrethroid for residual spraying, 24% (100/414 tonnes) for space spraying, and the remainder for treatment of nets and larviciding [7]. The exploitation of pyrethroids in China started from 1970s, and has been used throughout the country in order to control medically and agriculturally important arthropod pests, including mosquitoes. The area treated with pyrethroids occupies more than one third of the total insecticide-treated area in China [8]. It is critical that the susceptibility of malaria vectors to pyrethroids is preserved. Indeed, it has been recommended not to use pyrethroids for indoor residual spraying where there is high coverage with treated nets [9]. Pyrethroid resistance in malaria vectors has been mostly studied in the major African malaria vector, *Anopheles gambiae* [10-15]. High levels of resistance to pyrethroids have been reported in *An. sinensis* populations from China, Korea, and Mekong region (Vietnam, Cambodia and Laos) [8,16-21]. Resistance to insecticides can arise due to mutations in the insecticide target site (target site resistance), which in the case of pyrethroids is the para-type sodium channel gene, which is known as knockdown resistance (*kdr*), is caused by a single mutation in the S6 transmembrane segment of domain II in the voltage-gated sodium channel (VGSC) gene [22]. In recent years, *An. sinensis* in Henan Province has developed high degree of resistance to deltamethrin [23]. In the present study, the frequency of *kdr* mutations from *An. sinensis* was detected in Henan province, China.

## Methods

### Mosquito collection

Adult *An. sinensis* were captured from six different geographical sites in August 2013 in Henan province of China, including Kaifeng, Tongbai, Tanghe, Pingqiao, Shihe, and Yongcheng counties. The mosquitoes (carcasses/intact mosquitoes) were preserved individually in 1.5 ml microtubes for further molecular analyses.

### DNA extraction

Each mosquito was used for DNA extraction with the MaqExtractorTm Kit (Toyobo co. Ltd). Briefly, 1) each mosquito was placed at the bottom of a 1.5 ml microtubes. 2) 750 μl lysis and binding solution and 40 μl magnetic beads were added and mixed for 10 min by vortex. 3) Supernatant was removed by magnetic capture. 4) Magnetic beads were washed three times by 900 μl washing solution and 900 μl 70% ethanol respectively. 5) 100 μl sterilized water was added and well mixed for 10 minutes. 6) Supernatant was collected by magnetic capture and place in a fresh tube. 7) Extracted DNA was stored at −20°C for PCR.

### Molecular identification and detection of *kdr* mutation

Molecular identifications of *An. sinensis* species were conducted by using species-specific primers and amplification of the ITS2 [24]. To determine point mutations of the para-type sodium gene at positions 1014, a 325 bp fragment of the para-type sodium gene including position 1014 was amplified. PCR primers were designed based on the *An. sinensis* sequences of the DIIS6 region of the para-type sodium gene according to reference [25]. The allele-specific primers designed were: kdrF (5’- TGC CAC TCC GTG TGT TTA GA-3’) and kdrR (5’- GAG CGA TGA TGA TCC GAA AT -3’) in a reaction mixture (50 μl) that contained 1× Buffer, 1.5 mM of MgCl_2_, 200 μM of each dNTP, 0.5 μM of each primers and 0.625 unit of Taq DNA polymerase. The conditions of PCR were: an initial denaturation at 95°C for 5 min, followed by 35 cycles at 95°C for 30 S, 52°C for 30 S and 72°C for 30 S, and a final extension step at 72°C for 7 min. The PCR products were purified with QIAquick PCR purification kit (Qiagen) and direct sequencing was done at Sangon Biotech Inc. Primers used for sequencing were in both forward and reverse directions using the same primers.

### Statistical analysis

The *kdr* allele frequency was calculated in each population and statistical differences among populations were examined using the *χ*-test.

### Ethics statement

No specific permits were required for the described field studies. For mosquito collection, oral consent was obtained from field and house owners in each location. These locations were not protected land, and the field studies did not involve endangered or protected species.

## Results

### Molecular identification

348 Anopheles were captured in six counties including Kaifeng, Tongbai, Tanghe, Pingqiao, Shihe, and Yongcheng counties in Aug 2013. 208 Anopheles were received molecular identification, of which 169 (81.25%) were *An. sinensis*, 25 (12.02%) were *Anopheles yatsushiroensis*, and 12 (5.77%) were *Anopheles lesteri* (Table 1).

**Table 1:** **Molecular identification of Anopheles species in Henan province**

**County**	**Sample size**	***An. sinensis***	***An. yatsushiroensis***	***An. lesteri***
	**(n)**	**(% (n))**	**(% (n))**	**(% (n))**
Kaifeng	80	97.50 (78)	0.00 (0)	2.50 (2)
Tongbai	44	79.55 (35)	18.18 (8)	0.00 (0)
Tanghe	20	70 (14)	25.00 (5)	0.00 (0)
Pingqiao	27	55.56 (15)	44.44 (12)	0.00 (0)
Shihe	6	100.00 (6)	0.00 (0)	0.00 (0)
Yongcheng	31	67.74 (21)	0.00 (0)	32.26 (10)
Total	208	81.25 (169)	12.02 (25)	5.77 (12)

### *Kdr* gene sequencing

**Figure 1:**
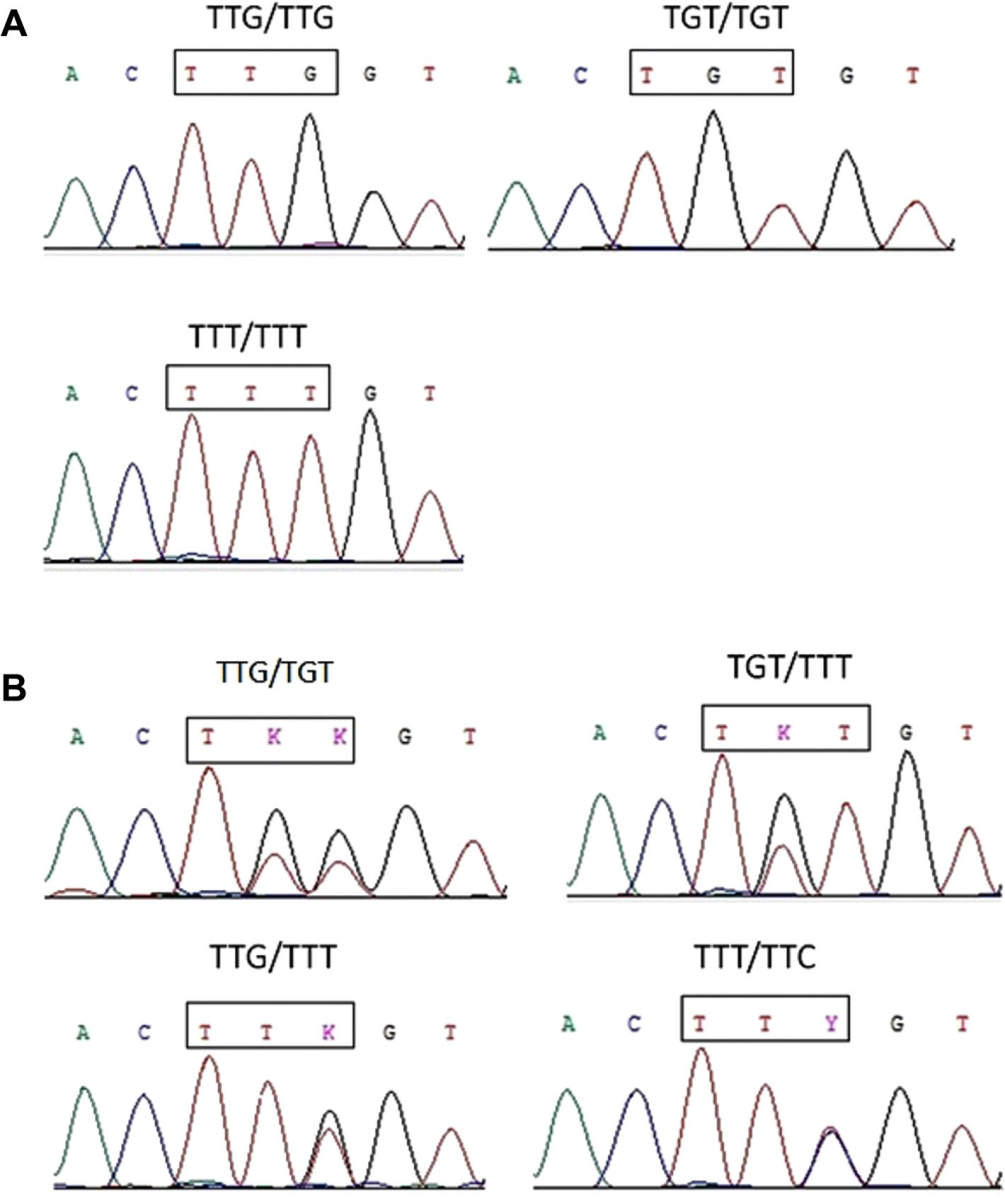
**Examples of nucleotide sequence chromatograms of** ***kdr*** **genotypes detected in** ***Anopheles sinensis*** **from Henan province.** The position at codon 1014 of the para-type sodium channel gene is indicated by a rectangle box. **A**: three types of homozygote genotypes detected; and **B**: four types of heterozygote genotypes detected (K = G/T; Y = T/C; S = G/C).

A 325 bp fragment of the para-type sodium channel gene including position 1014 was successfully sequenced from 139 Anopheles, of which 125 (89.93%) were *An. sinensis*, 12 (8.63%) were *An. yatsushiroensis*, two (1.44%) were *An. lesteri*. The molecular analyses revealed that mutations existed at codon 1014 in *An. sinensis* but not in *An. yatsushiroensis* and *An. lesteri*. The wild-type kdr codon sequence spanning position 1014 was TTG. Three types of *kdr* mutations were detected: two L1014F (TTT and TTC) lead to a change from Leucine to Phenylalanine, one L1014C (TGT) leads to a Leucine to Cysteine substitution. A total of seven genotypes were identified in the three populations. Three types of homozygote genotypes detected: TTG/TTG, TTT/TTT, TGT/TGT and four types of heterozygote genotypes detected: TTG/TTT, TTG/TGT, TGT/TTT, and TTT/TTC (GenBank accesion numbers: KF927155- KF927164) (Figure 1).

### Distribution of kdr allele frequencies in *An. sinensis* populations

Frequency of *kdr* mutation was 73.60% (92/125) from population of *An. sinensis* in Henan province, of which L1014F (TTT + TTC) allele frequencies accounted for 46.40% (58/125), and was higher than that of L1014C (TGT) which accounted for 27.20% (34/125) (*χ2* = 55.423, *P <* 0.001). No wild-type *kdr* sequence was found and frequency of *kdr* mutation from population of *An. sinensis* was 100.00% (49/49) in Kaifeng county. The L1014F (TTT + TTC) allele frequencies accounted for 73.47% (36/49), and was higher than that of L1014C (TGT) which accounted for 26.53% (13/49) in Kaifeng county (*χ*2 = 21.592, *P <* 0.001).

The frequency of *kdr* mutation in Kaifeng county was higher than that of 37.93% (11/29) in Tongbai county, 54.55% (6/11) in Pingqiao, 50.00% (3/3) in Shihe, and 62.50% (10/16) in Yongcheng county, respectively (*χ2* = 39.538, *P <* 0.001; *χ2* = 24.298, *P <* 0.001; *χ2* = 25.913, *P <* 0.001; *χ2* = 20.244, *P <* 0.001). The second high frequency of *kdr* mutation was found in Tanghe county, which was 92.86% (13/14), and was higher than that in Tongbai county (*χ2* = 11.550, *P* = 0.0018) (Table 2).

**Table 2:** **Frequency (% (n)) of** ***kdr*** **alleles in three** ***An. sinensis*** **population from Henan province**

**County**	**Sample size(n)**	**L1014 TTG**	**L1014C (TGT)**	**L1014F (TTT + TTC)**	**Population kdr mutation frequency (TGT + TTT + TTC + TGG)**
Kaifeng	49	0 (0)	26.53 (13)	73.47 (36)	100 (49)a
Tongbai	29	62.07 (18)	13.79 (4)	24.14 (7)	37.93 (11)
Tanghe	14	7.14 (1)	50.00 (7)	42.86 (6)	92.86 (13)b
Pingqiao	11	45.45 (5)	36.36 (4)	18.18 (2)	54.55 (6)
Shihe	6	50.00 (3)	16.67 (1)	33.33 (2)	50.00 (3)
Yongcheng	16	37.50 (6)	31.25 (5)	31.25 (5)	62.50 (10)
Total	125	26.40 (33)	27.20 (34)	46.40 (58)	73.60 (92)

Note: a. the frequency of *kdr* mutation in Kaifeng county was higher than that in Tongbai, Pingqiao, Shihe, and Yongcheng county (*P <* 0.001); b. the frequency of *kdr* mutation in Tanghe county was higher than that in Tongbai county (*P* = 0.001).

## Discussion

According to surveys conducted in 1930s, vivax malaria (temperate strains) was prevalent in Henan province of China [26]. There were two large epidemics of vivax malaria happened respectively in 1960s and at the beginning of 1970s. The incidence in the whole province was as high as 16,944.40/100 thousand in 1970 [27]. With active implementation of malaria control measures (integrated vector control measures and appropriate treatment of malaria cases) for more than 30 years, considerable success had been achieved and human cases infected with *Plasmodium vivax* have been reduced significantly in Henan Province in the end of 1980s, and malaria incidence was below 1/10,000 in most areas. By 1992, malaria had been nearly eliminated (incidence less than 1/100,000), with only 318 malaria cases observed in the province [28]. From 2000 to 2006, there was a substantial increase in malaria cases due to re-emerging vivax malaria in Huang-Huai plain. Dramatic vivax malaria resurgence appeared in Yongcheng and Xiayi county of east of Henan province in 2006, which were 307.04% and 360.94% higher than that in 2005 respectively. In Yongcheng county, malaria incidence was 1.23/10,000 in 2004 and 5.19/10,000 in 2005, which is 13 and 3.22 times higher than that of the previous year, respectively [29]. 36 malaria outbreaks and 1825 cases were found in four townships of Yongcheng county, accounted for 63.2% of total malaria cases of Henan province, the highest malaria incidence was up to 4.0% in a village with 43 malaria cases [30].

The degree of epidemicity of malaria is decided by many factors, of which vector efficiency is one of the most important ones. Ten Anopheles species were found in Henan province including *An. sinensis*, *Anopheles lindesayi*, *Anopheles koreicus*, *Anopheles kweiyangensis*, *Anopheles minimus*, *Anopheles pattoni*, *Anopheles maculates*, *Anopheles gigas baileyi*, *Anopheles anthropophagus*, and *An. yatsushiroensis*. The only two vector species of vivax malaria in Henan province are *An. sinensis* and *An. anthropophagus* [27] - *An. anthropophagus* and *An. lesteri* were same species. *Anopheles yatsushiroensis* has recently been declared a synonym of *Anopheles pullus* [31,32] and a possible vector of vivax malaria in Korea [33,34]. In this study, *An. yatsushiroensis* is the second most common *Anopheles* species in Pingqiao county (44.44%), after *An. sinensis*. The next step is to understand whether or not *An. yatsushiroensis* be a possible vector of vivax malaria and the pyrethroid susceptibility to this specie in Pingqiao.

Vector capacity of *An. anthropophagus* is higher than that of *An. sinensis,* but *An. sinensis* is the major transmitting vectors in the central provinces due to its widespread distribution [35,36]. The vectorial efficiency of *An. sinensis* increased during the warmest months from June to August of the year when people frequently sleep outdoor near their fields and unprotected by bed nets [37]. The re-emergence in the Huanghuai plain of central China, including the four provinces of Anhui, Henan, Hubei and Jiangsu were associated with the predominant vector *An. sinensis* [38], which also plays an important role in the maintenance of *P. vivax* malaria transmission [39]. Pan *et al.* investigated vectorial capacities of *An. sinensis* in 2007, the vectorial capacities of *An. sinensis* in Huaiyuan and Yongcheng county were 0.7740 and 0.5502, respectively [40]. The results showed that the vector capacity was about 2.3 and 1.7 times higher than 0.331 in the 1990s [41], and was 4.6 and 3.3 times higher than 0.1686 in Henan during 1996–1998 [42], respectively. It was considered that *An. sinensis* was the sole potential vector of *P. vivax* malaria in Yongcheng city of Henan province with a 2.78-fold vectorial capacity in 2010 (0.4689) compared to 0.1686 in the 1990s [43,44].

One of the most effective measures to prevent malaria transmission relies on vector control through the use of insecticides, primarily pyrethroids. The overdoses of pyrethroids poses strong selection pressure on mosquito populations for resistance, and have quickly led to the presence and spread of insecticide-resistant mosquitoes, which have caused serious problems for malaria-controlling interventions [34]. The results showed that the KT50 of *An. sinensis* to deltamethrin was 1122.50, 89.65, and 960 min in Tongbai, Huaibin and Yongcheng county, respectively. The mortality rate of *An. sinensis* in 24 h-post-exposure to 0.05% deltamethrin was 92.08%, 77.14%, and 63.46%, with a resistance degree of M, R and R, respectively. The results showed that *An. sinensis* has developed high degree of resistance to deltamethrin in Henan province [23]. The same results were found in Anhui, Hubei, Jiangsu and Shandong province of central China [19,45,46], and Zhejiang, Hainan, Guangxi and Hunan of south China [47-50], which suggested that pyrethroid resistance was already widespread in natural populations of China.

Knockdown resistance, which are mutations in the para-type sodium channel gene, the target site of pyrethroids is one major resistance mechanisms, which causing a change in affinity between the insecticide and its binding site that reduces sensitivity to the insecticide. In present study, *kdr* mutation *An. sinensis* from Henan province was examined. A high frequency (100.00%) of *kdr* mutations was found in populations from Kaifeng county, but not in populations from Tongbai county (37.93%), with the average frequency of 73.60% (92/125) in Henan province. The previous studies reported that the frequencies of the *kdr* allele of *An. sinensis* in China ranged from <10% to >85%, indicating a similar genetic outcome under selective pressure from insecticide treatment [20,21,50,51].

In this study, molecular analysis of *kdr* gene revealed that mutations at codon 1014 existed only in *An. sinensis*, whereas no *kdr* mutations were observed in *An. yatsushiroensis* and *An. lesteri*. Several mutations at codon 1014 of the *kdr* allele, such as L1014F (Leu-to-Phe), L1014S (Leu-to-Ser), and L1014C (Leuto- Cys) have been reported in many Anopheles species [52-55]. In this study, frequency of L1014F allele accounted for 46.40% (58/125), and was higher than that of L1014C which accounted for 27.20% (34/125) (*χ2* = 55.423, *P <* 0.001). The results suggested that L1014F mutation was a major allele that showed a high allele frequency, whereas L1014C mutation was a minor allele that showed a low allele frequency within the *An. sinensis* populations in Henan province.

A study using stepwise multiple regression analyses in mosquito populations from central China found that both *kdr* mutations and monooxygenase activity were significantly associated with deltamethrin resistance, with monooxygenase activity playing a stronger role [24]. The results suggest that different mechanisms of resistance could evolve in geographically different populations.

## Conclusions

The observed pyrethroid resistance and high *kdr* mutation frequency in populations of *An. sinensis* could profoundly affect the current malaria vector control programme in Henan province. The identification of widespread *kdr* mutations suggests that the development of reliable resistance surveillance tools is an important topic for future research. This needs an urgent call for implementing rational resistance management strategies and integrated vector control intervention.

## References

[1] Oh SS, Hur MJ, Joo GS, Kim ST, Go JM, Kim YH (2010). Malaria vector surveillance in Ganghwa-do, a malaria-endemic area in the Republic of Korea. Korean J Parasitol.

[2] Manh CD, Beebe NW, Van VN, Quang TL, Lein CT, Nguyen DV (2010). Vectors and malaria transmission in deforested, rural communities in north-central Vietnam. Malar J.

[3] Paredes-Esquivel C, Harbach RE, Townson H (2011). Molecular taxonomy of members of the *Anopheles hyrcanus* group from Thailand and Indonesia. Med Vet Entomol.

[4] Hii J, Rueda LM (2013). Malaria vectors in the Greater Mekong Subregion: overview of malaria vectors and remaining challenges. Southeast Asian J Trop Med Public Health.

[5] WHO (2014). WHO guidance for countries on combining indoor residual spraying and long-lasting insecticidal nets.

[6] WHO (2014). Review of current evidence on combining indoor residual spraying and long-lasting insecticidal nets.

[7] WHO (2011). Global Insecticide Use for Vector-Borne Disease Control: A 10-year assessment (2000–2009).

[8] Wang DQ, Xia ZG, Zhou SS, Zhou XN, Wang RB, Zhang QF (2013). A potential threat to malaria elimination: extensive deltamethrin and DDT resistance to Anopheles sinensis from the malaria-endemic areas in China. Malar J.

[9] WHO (2011). The technical basis for coordinated action against insecticide resistance: preserving the effectiveness of modern malaria vector control: meeting report.

[10] Okia M, Ndyomugyenyi R, Kirunda J, Byaruhanga A, Adibaku S, Lwamafa DK (2013). Bioefficacy of long-lasting insecticidal nets against pyrethroid-resistant populations of *Anopheles gambiae s.s.* from different malaria transmission zones in Uganda. Parasit Vectors.

[11] Nwane P, Etang J, Chouaibou UM, Toto JC, Koffi A, Mimpfoundi R (2013). Multiple insecticide resistance mechanisms in *Anopheles gambiae s.l.* populations from Cameroon, Central Africa. Parasit Vectors.

[12] Bigoga JD, Ndangoh DN, Awono-Ambene PH, Patchoke S, Fondjo E, Leke RG (2012). Pyrethroid resistance in *Anopheles gambiae* from the rubber cultivated area of Niete, South Region of Cameroon. Acta Trop.

[13] Kawada H, Dida GO, Ohashi K, Komagata O, Kasai S, Tomita T (2011). Multimodal pyrethroid resistance in malaria vectors, *Anopheles gambiae s.s., Anopheles arabiensis,* and *Anopheles funestus s.s.* in western Kenya. PLoS One.

[14] Dabire KR, Diabate A, Namountougou M, Toe KH, Ouari A, Kengne P (2009). Distribution of pyrethroid and DDT resistance and the L1014F kdr mutation in *Anopheles gambiae s.l.* from Burkina Faso (West Africa). Trans R Soc Trop Med Hyg.

[15] Kamau L, Agai D, Matoke D, Wachira L, Gikandi G, Vulule JM (2008). Status of insecticide susceptibility in *Anopheles gambiae* sensu lato and *Anopheles funestus* mosquitoes from western Kenya. J Insect Sci.

[16] Verhaeghen K, Van Bortel W, Trung HD, Sochantha T, Keokenchanh K, Coosemans M (2010). Knockdown resistance in *Anopheles vagus, An. sinensis, An. paraliae* and *An. peditaeniatus* populations of the Mekong region. Parasit Vectors.

[17] Kang S, Jung J, Lee S, Hwang H, Kim W (2012). The polymorphism and the geographical distribution of the knockdown resistance (kdr) of *Anopheles sinensis* in the Republic of Korea. Malar J.

[18] Chang KS, Yoo DH, Shin EH, Lee WG, Roh JY, Park MY (2013). Susceptibility and resistance of field populations of *Anopheles sinensis* (Diptera: Culicidae) collected from Paju to 13 insecticides. Osong Public Health Res Perspect.

[19] Chang XL, Xue YQ, Zhang AD, Zhu GD, Fang Q (2013). [Deltamethrin resistance, metabolic detoxification enzyme and *kdr* mutation in *Anopheles sinensis* in region along Huaihe River in Anhui Province](in Chinese). Chin J Schistosomiasis Control.

[20] Xu T, Zhong D, Tang L, Chang X, Fu F, Yan G (2014). *Anopheles sinensis* mosquito insecticide resistance: comparison of three mosquito sample collection and preparation methods and mosquito age in resistance measurements. Parasit Vectors.

[21] Qin Q, Li Y, Zhong D, Zhou N, Chang X, Li C (2014). Insecticide resistance of *Anopheles sinensis* and *An. vagus* in Hainan Island, a malaria-endemic area of China. Parasit Vectors.

[22] Hemingway J, Hawkes NJ, McCarroll L, Ranson H (2004). The molecular basis of insecticide resistance in mosquitoes. Insect Biochem Mol Biol.

[23] Liu Y, Chen JS, Zhou RM, Qian D, Chen QW, Xu BL (2012). [Investigation on the sensitivity of *Anopheles sinensis* to insecticide](in Chinese). Chin J Parasitol Parasit Dis.

[24] Yajun M, Xu J (2005). The Hyrcanus group of Anopheles (Anopheles) in China (Diptera: Culicidae): species discrimination and phylogenetic relationships inferred by ribosomal DNA internal transcribed spacer 2 sequences. J Med Entomol.

[25] Zhong D, Chang X, Zhou G, He Z, Fu F, Yan Z (2013). Relationship between knockdown resistance, metabolic detoxification and organismal resistance to pyrethroids in *Anopheles sinensis*. PLoS One.

[26] Tang LH, Gao Q. [Malaria control and eliminate in China.]. *Shanghai: Shanghai scientific & technical publishers* 2013; 10.

[27] Su SZ, Ma YX, Wang Z (1995). [Malaria study and control in Henan province].

[28] Xu BL, Su YP, Shang LY, Zhang HW (2006). Malaria control in Henan Province, People's Republic of China. Am J Trop Med Hyg.

[29] Zhang HW, Su YP, Zhou GC, Liu Y, Cui J, Wang ZQ (2007). [Re-emerging malaria in Yongcheng City of Henan Province.] (in Chines). Chin J Vector Biol Control.

[30] Zhou GC, Zhang HW, Su YP, Zhou SS, Huang F (2008). [Analysis of malaria outbreak in Yongcheng county of Henan province](in Chines). J Trop Med.

[31] Hwang UW, Yong TS, Ree HI (2004). Molecular evidence for synonymy of *Anopheles yatsushiroensis* and *An. pullus*. J Am Mosq Control Assoc.

[32] Park SJ, Choochote W, Jitpakdi A, Junkum A, Kim SJ, Jariyapan N (2003). Evidence for a conspecific relationship between two morphologically and cytologically different forms of Korean *Anopheles pullus* mosquito. Mol Cells.

[33] Shin EH, Kim TS, Lee HW, Lee JS, Lee WJ (2002). Vector competence of *Anopheles lesteri* Baisas and Hu (Diptera: Culicidae) to *Plasmodium vivax* in Korea. Korean J Parasitol.

[34] Ree HI (2005). Studies on Anopheles sinensis, the vector species of vivax malaria in Korea. Korean J Parasitol.

[35] Chen JS, Su YP, Liu H, Su YJ (2001). [Apply of mathematic epidemiological in malaria analysis in South of Henan province] (in Chinese). Henan Preventive Med.

[36] Gu ZC, Shang LY, Chen JS, Zheng X, Su YJ, Li AM (2001). [The role of *Anopheles anthropophagus* in malaria transmission in in Xinyang City of Henan Province] (in Chinese). Chin J Parasitol Parasit Dis.

[37] Qunhua L, Xin K, Changzhi C, Shengzheng F, Yan L, Rongzhi H (2004). New irrigation methods sustain malaria control in Sichuan Province, China. Acta Trop.

[38] Zhou SS, Huang F, Wang JJ, Zhang SS, Su YP, Tang LH (2010). Geographical, meteorological and vectorial factors related to malaria re-emergence in Huang-Huai River of central China. Malar J.

[39] Liu XB, Liu QY, Guo YH, Jiang JY, Ren DS, Zhou GC (2011). The abundance and host-seeking behavior of culicine species (Diptera: Culicidae) and *Anopheles sinensis* in Yongcheng city. People's Republic of China. Parasit Vectors.

[40] Pan JY, Zhou SS, Zheng X, Huang F, Wang DQ, Shen YZ (2012). Vector capacity of *Anopheles sinensis* in malaria outbreak areas of central China. Parasit Vectors.

[41] Qian HL, Tang LH, Tang LY (1996). [Preliminary estimation on the critical value of man biting rate and vectorial capacity of *Anopheles sinensis*.] (in Chinese). Practical Preventire Med.

[42] Qu CZ, Su TZ, Wang MY, Dong T, Shi CM, Zhang RG (2000). [Vectorial capacity of malaria transmission of *Anopheles sinensis* in Zhengzhou in nature.] (in Chinese). J Henan MedUniv.

[43] Liu XB, Liu QY, Guo YH, Jiang JY, Ren DS, Zhou GC (2012). Random repeated cross sectional study on breeding site characterization of *Anopheles sinensis* larvae in distinct villages of Yongcheng City. People's Republic of China. Parasit Vectors.

[44] Zhou SS, Wang Y, Li Y (2011). [Malaria situation in the People's Republic of China in 2010] (in Chinese). Chin J Parasitol Parasit Dis.

[45] Li JL, Zhou HY, Cao J, Zhu GD, Wang WM, Gu Y (2011). [Sensitivity of *Anopheles sinensis* to insecticides in Jiangsu Province] (in Chinese). Chin J Schistosomiasis Control.

[46] Yu PH, Zhang HX, Zhang SQ, Xu BZ (2000). [Survey of susceptibility of anopheline vectors to insecticides in a malaria mesoendemic area, Hubei Province] (in Chinese). Chin J Parasitol Parasit Dis.

[47] Zeng LH, Wang SQ, Sun DW, Zhao W, Li SG, Yang X (2011). [Resistance assay of malaria vectors to four kinds of common insecticides in some endemic areas of Hainan Province] (in Chinese). Chin J Parasitol Parasit Dis.

[48] Wang J (1999). Resistance to two pyrethroids in *Anopheles sinensis* from Zhejiang, China. J Am Mosq Control Assoc.

[49] Wang J (2000). Resistance and response to selection to deltamethrin in *Anopheles sinensis* from Zhejiang, China. J Am Mosq Control Assoc.

[50] Tan WL, Li CX, Wang ZM, Liu MD, Dong YD, Feng XY (2012). First detection of multiple knockdown resistance (kdr)-like mutations in voltage-gated sodium channel using three new genotyping methods in *Anopheles sinensis* from Guangxi Province, China. J Med Entomol.

[51] Bai L, Zhu GD, Tang JX, Zhang C, Liu YB, Li JL (2013). [Study on TaqMan-MGB real-time fluorescence quantitative PCR to detect gene mutation of kdr from *Anopheles sinensis*] (in Chinese). Chin J Schistosomiasis Control.

[52] Dabire RK, Namountougou M, Diabate A, Soma DD, Bado J, Toe HK (2014). Distribution and frequency of kdr mutations within *Anopheles gambiae* s.l. populations and first report of the ace.1 G119S mutation in *Anopheles arabiensis* from Burkina Faso (West Africa). PLoS One.

[53] Singh OP, Dykes CL, Lather M, Agrawal OP, Adak T (2011). Knockdown resistance (kdr)-like mutations in the voltage-gated sodium channel of a malaria vector *Anopheles stephensi* and PCR assays for their detection. Malar J.

[54] Singh OP, Dykes CL, Das MK, Pradhan S, Bhatt RM, Agrawal OP (2010). Presence of two alternative kdr-like mutations, L1014F and L1014S, and a novel mutation, V1010L, in the voltage gated Na + channel of *Anopheles culicifacies* from Orissa. India. Malar J.

[55] Luleyap HU, Alptekin D, Kasap H, Kasap M (2002). Detection of knockdown resistance mutations in *Anopheles sacharovi* (Diptera: Culicidae) and genetic distance with *Anopheles gambiae* (Diptera: Culicidae) using cDNA sequencing of the voltage-gated sodium channel gene. J Med Entomol.

